# Pathogenic mechanisms and therapeutic potential of the microbiome in premature ovarian insufficiency

**DOI:** 10.3389/fimmu.2025.1734367

**Published:** 2025-12-16

**Authors:** Shuai Ma, Lianwen Zheng, Xiaohua Zhuang, Min Wang, Yinggang Zou

**Affiliations:** 1 Department of Obstetrics and Gynecology, The Second Hospital of Jilin University, Changchun, China; 2 Department of Nephrology, The Second Hospital of Jilin University, Changchun, China

**Keywords:** etiology, fecal microbiota transplantation, gut microflora, human microbiome, infertility, metabolomics, primary ovarian insufficiency, therapeutic

## Abstract

The postponement of childbearing age has become a global issue. Factors such as increased work pressures on women and environmental changes have led to a rising incidence and younger onset of premature ovarian insufficiency (POI). POI not only impacts patients’ reproductive function but also heightens the risk of depression, anxiety, cognitive decline, premature mortality, osteoporosis, and cardiovascular disease. Exploring effective prevention and treatment strategies for POI can slow ovarian ageing and safeguard female reproductive health. Microbiome research confirms that most human tissues and organs form dynamic, interactive systems with symbiotic microbes that play a crucial role in female reproductive function. Previous studies on the microbiome and female reproductive health have rarely focused on POI. The proposed ‘Microbiota-Ovary Axis’ aims to establish an integrated regulatory framework. This theoretical model systematically elucidates how microbial signals influence ovarian function through four core pathways: the hypothalamic-pituitary-ovarian (HPO) axis, metabolism and endocrine regulation, immunoregulation, and oxidative stress. Evaluating the efficacy of dietary modifications, probiotics, and microbiota transplantation in animal models and preliminary clinical studies will establish a robust theoretical foundation for developing microbiota-targeted innovative diagnostic and therapeutic strategies for POI, thereby enhancing reproductive health throughout the female lifespan.

## Graphical Abstract

**Figure:**
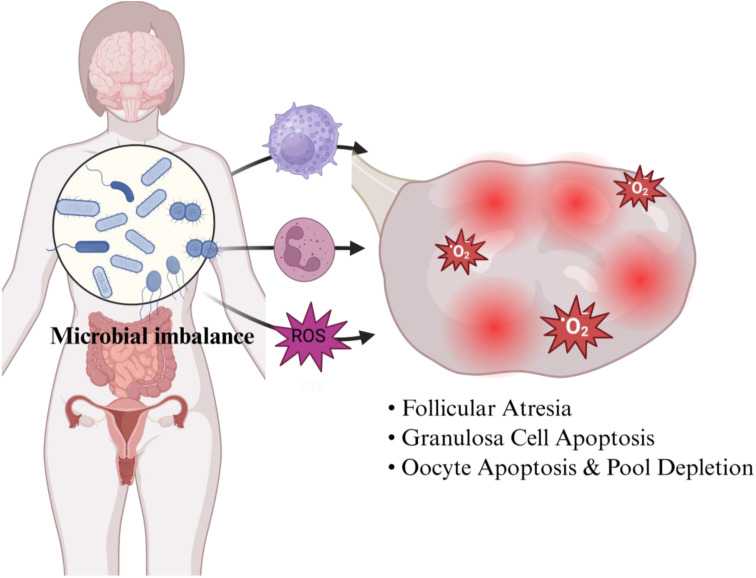

## 1 Introduction

Premature ovarian insufficiency (POI) denotes the occurrence of follicular depletion, insufficient sex hormone secretion, and diminished ovarian reserve in women under 40 years of age, resulting in impaired ovarian function (1). POI further leads to infertility and premature menopausal symptoms, causing significant psychological distress and social role pressures for patients (2). It also markedly increases the risk of long-term chronic conditions such as osteoporosis, cardiovascular disease, and cognitive decline, imposing a substantial burden on individuals and public health systems (3, 4). The etiology and pathogenesis of POI are complex, with current research suggesting associations with chromosomal abnormalities, surgical trauma, infections, autoimmune factors, and genetic mutations (5, 6). To date, approximately 50% of cases remain unexplained, categorized as idiopathic POI, highlighting substantial gaps in understanding its underlying mechanisms (7). For decades, reproductive medicine adhered to the traditional paradigm of the ‘sterile uterus’, viewing the female upper reproductive tract as a microenvironment that is sterile in health. However, innovations such as high-throughput sequencing and germ-free animal models have fundamentally overturned this understanding: the entire reproductive tract constitutes a continuous microbial continuum, with the proportion of lactobacilli within the corresponding microbiota gradually decreasing as one moves anatomically upwards (8). Emerging evidence not only confirms the presence of a functionally distinct microbial community within the female reproductive system but also reveals a significant association between reproductive tract dysbiosis and impaired ovarian function (9). The human microbiome refers to the collective sum of microorganisms and their genetic material residing within the human body, playing crucial roles in immunity and metabolism (10, 11). The complex regulatory network formed by the hypothalamic-pituitary-ovarian (HPO) axis and the Microbiota-Ovary Axis may play a crucial role in ovarian physiology and pathology (12). Microbial communities may directly or indirectly induce ovarian dysfunction through mechanisms such as regulating inflammatory mediators, local immune homeostasis, influencing metabolite profiles, and modulating oxidative stress, thereby precipitating POI (13). Microbial communities may directly or indirectly induce ovarian dysfunction through mechanisms such as regulating inflammatory mediators, local immune homeostasis, influencing metabolite profiles, and modulating oxidative stress, thereby precipitating POI.

## 2 Overview of POI

The core of female reproductive health lies in normal ovarian function (14). Impairments in follicular development, excessive depletion, significant hormonal fluctuations, ovarian surgery, and radiotherapy or chemotherapy can lead to POI, posing a serious threat to women’s physical and mental well-being (15, 16). POI is a common reproductive disorder. According to the 2016 European Society of Human Reproduction and Embryology guidelines, physicians diagnose POI when a woman under 40 experiences oligomenorrhoea or amenorrhoea for over four months and has two follicle-stimulating hormone (FSH) measurements exceeding 25 U/L, taken at least four weeks apart (17). The etiology of POI may involve genetic factors, metabolic disorders, immune dysfunction, chemical damage, and environmental influences (18, 19). Current treatment primarily relies on hormone replacement therapy (HRT), which carries significant risks of adverse effects, including recurrence after discontinuation and increased long-term incidence of endometrial cancer, breast cancer, and cardiovascular disease (20). HRT essentially constitutes a symptomatic rather than curative strategy, incapable of reversing or restoring ovarian function once depleted, nor can it effectively address patients’ core concern—the loss of fertility (21). Nearly half of POI cases remain idiopathic, suggesting the existence of unknown key pathological mechanisms (22, 23).

As research advances, the microbiome’s functions extend far beyond initial expectations. Operating as a dynamic endocrine and immune organ, it profoundly influences ovarian physiological and pathological processes (24, 25). Microbiome balance is closely associated with POI and holds significant importance for maintaining female reproductive health. Researchers need to further investigate the specific mechanisms by which microbiome homeostasis influences POI to identify personalized microbiome therapeutic strategies based on multi-omics approaches. Microbiology could provide novel directions for clinical interventions to reverse or delay POI (26).

## 3 Microbiomics

The human microbiome refers to the microbial communities inhabiting both inside and outside the human body, encompassing bacteria, fungi, viruses, and other microorganisms distributed across various sites, including the oral cavity, skin, gut, reproductive tract, and brain. The collective genome of these microorganisms is termed the microbiome (27). The human microbiome far exceeds a simple collection of bacteria; it essentially functions as an ‘organ’ – a dynamic, functionally active system with systemic endocrine and immune regulatory capabilities (28). This vast ecosystem continuously generates and releases numerous bioactive small-molecule metabolites via intricate metabolic networks, including short-chain fatty acids (SCFAs), secondary bile acids (BA), and tryptophan derivatives (29). These microbial metabolites either enter the systemic circulation directly or exert distal regulation of host physiological processes via signaling pathways, such as G protein-coupled receptors (GPCRs) (30). Microbial metabolites not only locally shape or enhance the host immune system to maintain immunological equilibrium, but also directly intervene in endocrine function, influencing hormone synthesis, metabolism, and signaling pathways, thereby contributing to the pathogenesis of diseases across multiple human systems (31, 32). Research indicates that in adult male mice fed a high-fat diet, dysbiosis impacts the production of the seminal microbiome, subsequently affecting semen quality (33, 34). Findings by Mayneris Perxachs J et al. suggest dysbiosis may play a pivotal role in the pathogenesis of androgen-dependent metabolic disorders (35). Research indicates that mice in dysbiotic groups can reverse bone loss through microbiota repopulation, with microbial homeostasis affecting bone density through complex mechanisms, including alterations in osteoclast factor production and hormonal levels (36).

### 3.1 Intestinal microbiomics

The gut microbiome constitutes a complex microbial ecosystem. As a vital component of the microecological system, the gut microbiota (GM) resides within both humans and animals (37). The human gut harbors over 1,000 distinct bacterial species, totaling more than 100 trillion (38). Owing to its vast quantity and potent functions, researchers often refer to the GM as the human body’s ‘second genome”. Under normal conditions, the GM maintains a dynamic equilibrium influenced by both the host and external factors, with its effects on the host varying according to changes in the host environment. Within the GM, approximately 90% of the bacteria belong to the phyla Firmicutes and Bacteroidetes, followed by Actinobacteria and Proteobacteria (39). The GM participates in multiple physiological processes, including the synthesis of SCFAs and vitamins, as well as the metabolism of lipids and BA. The GM and its metabolites form the cornerstone of the intestinal chemical barrier (40). Metabolic products generated by the GM are crucial for immune regulation and anti-inflammatory functions, playing an essential role in maintaining intestinal homeostasis while effectively preventing harmful substances, such as bacteria and endotoxins, from entering the bloodstream and other tissues and organs (41). Disruption of the dynamic equilibrium of GM readily leads to compromise of the intestinal mucosal barrier, allowing endotoxins and lipopolysaccharides (LPS) to be released into the systemic circulation, activating inflammatory pathways and triggering the production of numerous inflammatory mediators, disrupting immune and metabolic homeostasis, and causing dysfunction of the reproductive system (42).

GM plays a significant role in female reproductive health (43). Menopause-related gut microbiome changes were associated with adverse cardiometabolic risk in postmenopausal women, indicating that the gut microbiome contributes to changes in cardiometabolic health during menopause (44).SCFAs regulate the expression of key genes in ovarian cells by inhibiting histone deacetylase activity (45). Santos-Marcos JA et al. observed that premenopausal women exhibited higher Firmicutes/Bacteroidetes ratios, greater relative abundances of Lachnospira and Roseburia, and elevated plasma glucagon-like peptide-1 (GLP-1) levels compared to postmenopausal women, whose plasma GLP-1 levels resembled those of males (46). Yan et al. demonstrated that zearalenone alters the composition and abundance of GM, including Bacteroidetes, Proteobacteria, Firmicutes, and Actinobacteria, with GM serving as a key mediator of zearalenone’s toxic effects on ovarian development (47). Xu et al. used GM from young mice to remodel GM in mice with reproductive ageing. They discovered that the young mouse GM altered serum inflammatory factor levels in aged mice and improved ovarian function by enhancing fertility, reducing follicular atresia and apoptosis, and increasing granulosa cell proliferation (48).

### 3.2 Microbiomics of the female reproductive tract

**Figure 1:**
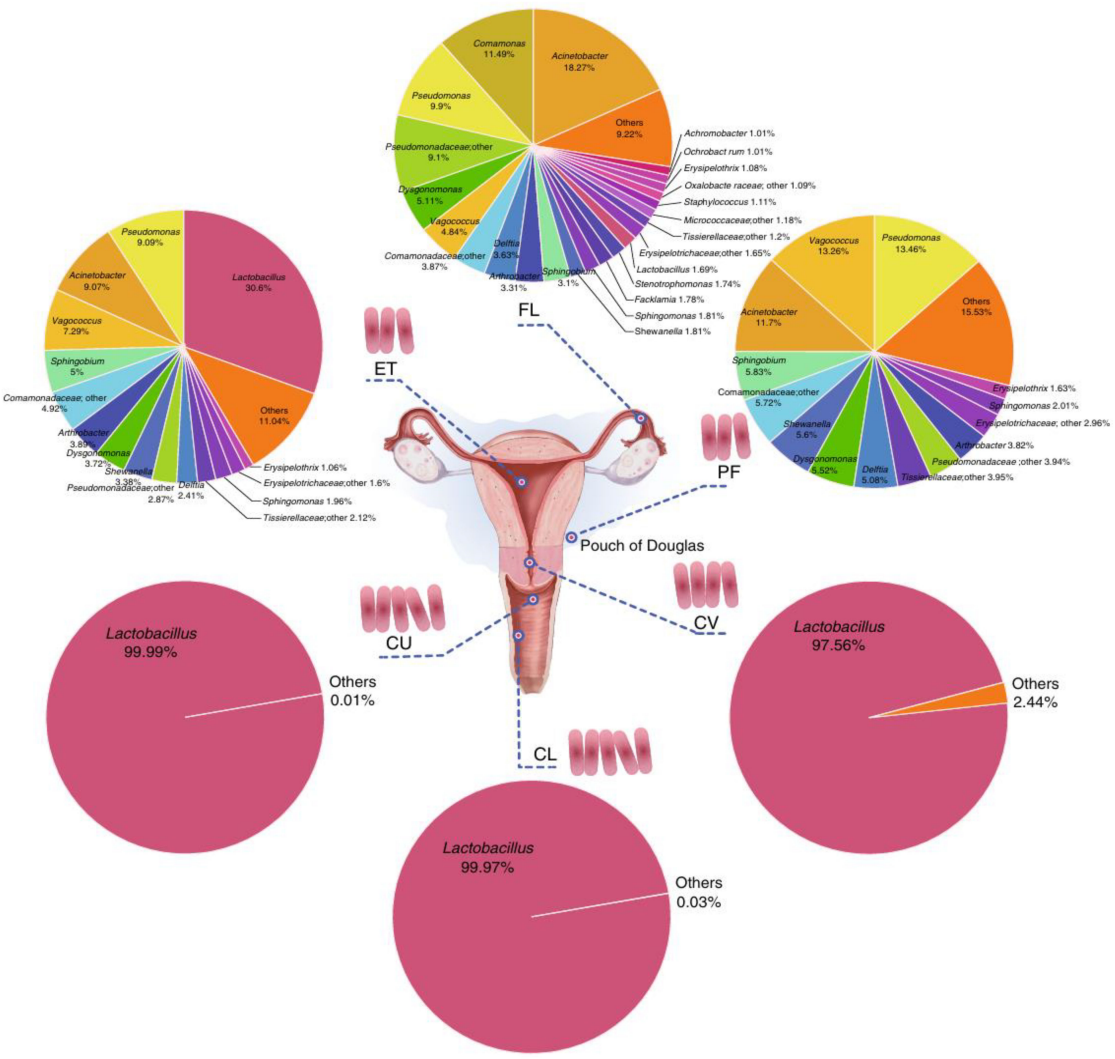
Composition of the vagino-uterine microbiota (8).

The female reproductive tract (FRT) microbiota is a vital component of the human microbiome, and alterations in this ecosystem are a significant factor influencing reproductive health (49). The FRT is divided into the upper and lower reproductive tracts, with the upper tract encompassing the cervical canal, uterine body, fallopian tubes, and ovaries. In contrast, the lower tract comprises the vagina and cervical os. Continuous microbial colonization exists throughout the entire FRT, and it has been established that the upper FRT is not a “sterile” environment (50) **Figure 1** (8). Bacterial counts in the upper tract are substantially lower than in the vagina, yet bacterial diversity within the upper tract microbiome exceeds that of the vaginal microbiota (51). The microbial composition of the FRT is relatively more straightforward than that of other body sites. The reproductive tract microbiome maintains a dynamic equilibrium through mutual regulation and coordination with the host and environment, playing a vital role in preserving female reproductive health. The female vaginal microbiota undergoes dynamic changes, with distinct dominant bacterial communities prevailing across different age groups. The FRT microbiome is influenced by multiple factors, including ethnicity, environment, menstrual cycle, sexual activity, and medication (52). Among these, hormonal fluctuations represent the most significant determinant, with pronounced shifts in microbial communities occurring during adolescence, menstruation, pregnancy, and menopause (53). The increase in endogenous steroid hormones during puberty correlates with greater diversity in the vaginal microbiota of adult women. During perimenopause, declining ovarian function and a sharp decrease in sex hormone levels coincide with dramatic changes in the vaginal microbiota, including a reduction in the relative abundance of Lactobacillus species (54). Consequently, disruptions in the reproductive tract microbiota exert significant impacts on reproductive health (55).

## 4 Microorganism ovary axis

The “microbiome-ovarian axis” represents a novel and dynamic theoretical framework. The microbiota exhibits correlations with female reproductive health, exerting a significant influence upon it (56). Dysbiosis leads to reduced SCFAs while increasing pathogen-associated molecular patterns such as LPS. SCFAs elevate estrogen levels; prolonged hyperestrogenism increases the risk of conditions including endometriosis, complex endometrial hyperplasia, and endometrial carcinoma (57). Microbiome dysbiosis shifts the immune balance toward a pro-inflammatory phenotype, elevating circulating levels of pro-inflammatory cytokines. These inflammatory mediators circulate to the ovaries. Upon receiving these abnormal signals, the ovaries’ resident immune cells become activated. They present self-antigens via major histocompatibility complex (MHC) II molecules while simultaneously secreting increased local inflammatory factors, recruiting and activating cytotoxic T cells. Interleukin (IL)-1β and tumor necrosis factor-α (TNF-α) directly induce granulosa cell apoptosis, suppress aromatase activity, and disrupt the estrogen synthesis microenvironment. Cytokines such as interferon-gamma (IFN-γ) upregulate the Fas/FasL apoptosis pathway, accelerating follicular atresia. Microbiota metabolites influence sex hormone-binding globulin levels, indirectly regulating androgen bioavailability. Dysbiosis generates excessive reactive oxygen species (ROS) and inflammatory mediators, altering intestinal mucosal permeability and increasing pathways for LPS entry into the systemic circulation, further affecting blood glucose levels and insulin secretion (58). Disruptions in androgen and insulin levels interfere with follicular maturation and development (59).

On the one hand, the proposal of the FRT axis suggests that metabolites produced by the microbiota modulate the FRT-brain axis (60). Research has demonstrated the existence of vaginal-brain and uterine-brain axes, such as the gonadotropin-releasing hormone (GnRH) axis, which stimulates pituitary secretion of luteinizing hormone (LH) and FSH, thereby increasing ovarian estrogen production. Conversely, the gut-gonadal axis links the intestinal tract and the reproductive system. GM and its metabolites influence reproductive function through multiple mechanisms, including regulation of the HPO axis, endocrine-metabolic homeostasis, immune balance, alleviation of oxidative stress, and improvement of inflammatory states (61). Zheng et al., using D-galactose-induced POI mice as a model, discovered that Lycium barbarum polysaccharides (LBP) could ‘awaken’ dormant ovaries: total follicle count and follicle numbers across all stages significantly rebounded, with concurrent decreases in FSH and LH. Disrupted oestrous cycles normalized, leading to increased litter sizes. Further 16S rRNA sequencing revealed that LBP remodeled the GM, enriching key genera, including Faecalibaculum, Bilophila, and Anaerofustis. Metabolome-microbiome analysis further suggested that LBP’s follicle-stimulating effect is mediated via the gut-gonadal axis (62). Microbiome science offers novel insights into understanding the etiology and mechanisms of POI. The microbiota participates in the pathogenesis of reproductive endocrine disorders through pathways that include neurotransmitter synthesis, regulation of sex hormones, stimulation of inflammatory factor production, and influence on immune function and metabolic homeostasis (63).

## 5 Microbial imbalance in POI

### 5.1 Imbalance of GM

**Figure 2:**
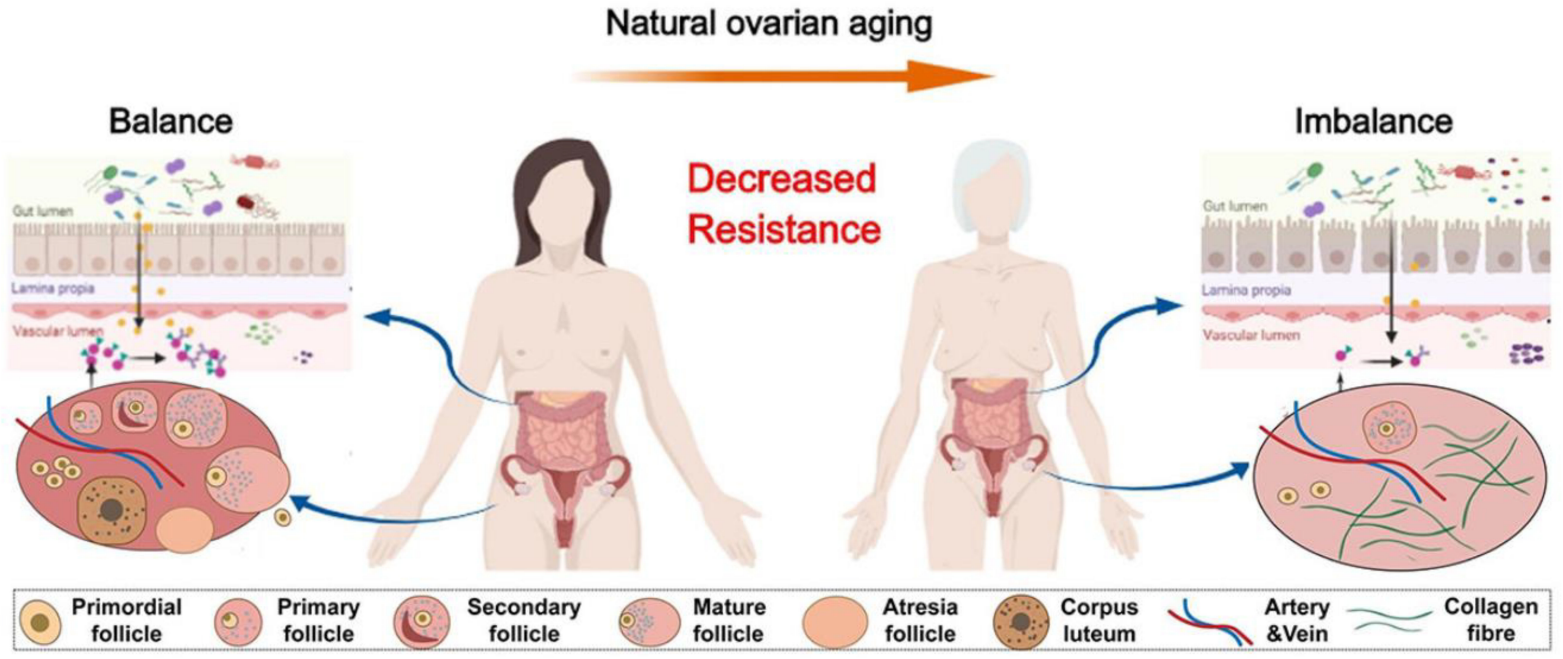
The physiologic succession of gut microbiota across natural ovarian aging (64).

GM is closely associated with the onset and progression of POI. **Figure 2** (64). Beyond its links to inflammation and autoimmune disorders, POI is also implicated in dyslipidaemia, suggesting that it may represent not merely a local ovarian pathology but a systemic metabolic disorder intrinsically linked to the GM. Research by Wang et al. indicates that alterations in the GM may exert a causal influence on POI risk, providing compelling evidence for the association between the GM and ovarian health (65). Compared to individuals with normal ovarian reserve function, patients with premature ovarian failure (POF) exhibit significant differences in the diversity, abundance, and composition of their GM. Cao et al. observed markedly increased α-diversity in the GM of POF mice, alongside elevated abundances of Clostridium XIVa and Bacteroides polymorpha, while Faecalibacterium and Helicobacter pylori abundances decreased (66). Wu et al. revealed substantial compositional differences between POI patients and healthy women. In POI patients, Bacteroidetes, Gardnerella, and Lactobacillus were markedly increased, while Firmicutes and Faecalibacterium were significantly reduced. These alterations may correlate with POI’s pathological mechanisms. Concurrently, GM changes were found to correlate with anti-Müllerian hormone (AMH), FSH, β-estradiol (E2), LH levels, and the FSH/LH ratio (67). Alterations in the GM of POI women were closely associated with FSH, LH, E2, AMH, and the FSH/LH ratio (68). Compared to healthy postmenopausal women, menopausal syndrome patients exhibited higher FSH and LH levels alongside markedly distinct GM compositions. Zhao et al. observed that premenopausal women exhibited significantly greater α-diversity than postmenopausal women, whereas POI patients showed reduced β-diversity. Further analysis at the phylum level revealed lower Firmicutes abundance and higher Bacteroidetes abundance in POI patients. At the genus level, POI patients showed higher abundances of Bacteroides butyricola and the Dorea genus than healthy women (69).

### 5.2 Imbalance of the reproductive microbiota

Research into the FRT microbiota encompasses studies on the structure and function of this microbial community. SCFAs produced by reproductive tract bacteria are present on the mucosal surfaces of female reproductive organs. SCFAs participate in steroid hormone synthesis through interactions with the lipoprotein CD36 and GPCRs in granulosa cells (70). In mouse models, LPS treatment was found to alter FSH and LH secretion while disrupting hormone receptors on ovarian and embryonic surfaces. *In vitro* analysis of peptidoglycans in Gram-positive bacterial cell walls demonstrated inhibition of progesterone (P) and androstenedione secretion, along with reduced expression of steroid synthases. Disruption of the vaginal microbiota may impair oocyte quality by altering the production of sex hormones, including steroids, thereby influencing the HPO axis. Alternatively, it may activate the immune system, leading to increased local inflammatory mediators and triggering chronic systemic inflammation via the blood and lymphatic systems, indirectly affecting oocyte development.

Compared to healthy subjects, POF patients exhibit distinct reproductive tract microbiota. Metabolic dysregulation and heightened immune responses within the reproductive tract of POF patients underlie microbial community imbalance (71). By comparing vaginal swab samples from infertile women and healthy women, Babu G et al. identified that the dominant genera in the vaginal microbiota of healthy women were Lactobacillus, Micrococcus, Enterococcus, and Staphylococcus. In contrast, the dominant microorganisms in the vaginal microbiota of infertile women were Candida and Enterococcus, with increased abundance of bacterial vaginosis-associated flora. Samples dominated by Lactobacillus were scarce, and the proportion of infertile women diagnosed with asymptomatic vaginitis was higher than that of healthy women (72).

#### 5.2.1 Vaginal microbiota

The vaginal microbiota refers to the local ecosystem formed by vaginal microorganisms (73). The stability of the vaginal microbiota is maintained collectively by its anatomical structure, microbial flora, the body’s endocrine system, and local immunity (74). Estrogen, local pH, and lactobacilli play crucial roles, interacting to sustain the dynamic equilibrium of vaginal microbial composition (75). Estrogen levels most significantly influence the vaginal microbiota (76). Elevated estrogen promotes maturation and proliferation of vaginal epithelial cells, while glycogen is converted into lactic acid by lactobacilli, thereby sustaining the vagina’s acidic environment (77). Based on microbial composition and abundance, Ravel et al. categorized the vaginal microbiota of women of reproductive age into five distinct community state types (CST). CST I, CST II, CST III, CST IV, and CST V are characterized by dominant populations of Lactobacillus crivulatus, Lactobacillus garnerii, Lactobacillus paracasei, diverse anaerobic bacteria, and Lactobacillus jensenii, respectively (78). The vaginal microbiota composition of the vast majority of healthy women of reproductive age most commonly comprises CST I, II, or V. Lactobacillus, as the primary resident bacteria in the normal female vagina, is central to maintaining vaginal microecological equilibrium (79). Lactobacilli form a physical barrier on the vaginal mucosal surface, effectively preventing direct contact between other microorganisms and the vaginal mucosa. Simultaneously, they break down glycogen within vaginal squamous epithelial cells into lactic acid, establishing a weakly acidic local vaginal environment that effectively inhibits the overgrowth of pathogenic bacteria (80). The bacteriocins, bacteriocin-like substances, hydrogen peroxide, and metabolic by-products secreted by lactobacilli impede the growth of pathogenic bacteria. Furthermore, they exert immunomodulatory effects by regulating the expression of immune factors and cytokines, thereby maintaining the equilibrium of the vaginal microecological environment.

The stability of the vaginal microbiome is pivotal for maintaining reproductive tract health (81). Disruption of the vaginal microbiota compromises the immune system, allowing exogenous bacteria and viruses to enter the vagina or enabling pre-existing pathogens to proliferate excessively and become dominant, thereby causing local microecological imbalance and subsequently leading to disease onset and progression (82). During oogenesis, vaginal microecological homeostasis may play a significant role in maintaining female oocyte quality. Research indicates that, compared with healthy controls, women with POF exhibit altered vaginal microbiota, characterized by reduced Lactobacillus species and increased Actinobacteria species (83). Wen et al. observed that while α-diversity of vaginal microbiota in POF patients under 40 years old did not differ from that of normal women, β-diversity exhibited significant alterations. Elevated serum FSH and LH levels in POF patients correlated positively with Actinobacteria, Gardnerella, and Bacteroides, and negatively with Firmicutes, Lactobacillus, and Bifidobacterium (84). Thus, vaginal microbiota imbalance is closely associated with diminished ovarian reserve.

#### 5.2.2 Cervical microbiota

The cervical microbiota constitutes a core component of the cervical microbiome. The cervical microbiota resembles the vaginal microbiota, dominated by lactobacilli. The predominant types are identical to those in the vaginal lactobacilli—Lactobacillus curvatus and Lactobacillus paracasei—though the cervical microbiota harbors a greater diversity of bacteria and viruses. Imbalance in the cervical microbiome is characterized by the displacement of lactobacilli from their dominant position. This disruption triggers the proliferation of endogenous pathogens and reduces resistance to exogenous pathogens originating outside the reproductive tract. Chen et al. analyzed vaginal, cervical, and intrauterine microbiota in women of reproductive age and found that cervical microbial composition resembled that of the vagina. However, the proportion of lactobacilli progressively decreased. Unlike the lactobacillus-dominated vaginal microbiota, no signature taxa were identified in the cervical canal or endometrium, suggesting these sites may serve as transitional zones between vaginal and upper genital tract microbiota (8). Winters et al. conducted 16S rRNA gene sequencing on cervical swabs from women, identifying Acinetobacter, Pseudomonas, Cloacibacterium, and Comamonadaceae as the most prevalent microorganisms in the cervix (85). Cervical microbiota detection may be subject to sampling bias influenced by vaginal microbiota, and subjective variations exist in research techniques and data analysis. Consequently, more scientifically rigorous protocols for detecting cervical microbiota require development.

#### 5.2.3 Uterine microbiota

With the advent of sequencing technology, the microbial communities residing in the uterine cavity have been gradually uncovered, confirming the distinctiveness, low abundance, and diversity of the endometrial microbiota. This discovery has overturned the “sterile uterus” hypothesis. Mitchell CM et al. identified Lactobacillus paracasei as the most prevalent bacterial genus within the uterine cavity, followed by Prevotella and Lactobacillus curvatus. They further observed that bacterial biomass within the uterine cavity was markedly lower than that within the vagina (86). Kyono et al. observed that the relative abundance of Lactobacillus in the endometrium and vagina of healthy women was significantly higher than in infertile patients. They hypothesized that increasing endometrial Lactobacillus levels might improve implantation outcomes in non-Lactobacillus-dominant infertility patients, suggesting that restoring a Lactobacillus-dominant endometrial environment through probiotic supplementation could facilitate pregnancy (87). Moreno et al. analyzed vaginal and endometrial microbiota in women achieving pregnancy via *In Vitro* Fertilisation and Embryo Transfer (IVF-ET), revealing Lactobacillus dominance in both vaginal and uterine cavities. Further investigation demonstrated that non-Lactobacillus-dominant microbiota trigger endometrial inflammatory responses. Inflammatory mediators impede blastocyst adhesion to the endometrium and correlate significantly with reduced implantation, pregnancy, ongoing pregnancy, and live birth rates. In studies of endometrial fluid in women of reproductive age, Lactobacillus species were similarly identified as the most abundant bacterial group. Crucially, the microbial community structure within endometrial fluid was found to be independent of regulation by female sex hormones (88).

#### 5.2.4 Tubal microbiota

Due to sampling difficulties, research on the fallopian tube microbiome remains relatively scarce (89). In the absence of infection, women’s fallopian tubes harbor detectable, diverse microbial communities influenced by hormonal fluctuations. Compared to the vagina, the fallopian tubes and ovaries harbor more diverse microbial populations that thrive under mildly alkaline conditions, contrasting with the acidic environment of the vagina. Miles et al. observed that the microbial compositions of the uterine myometrium, endometrium, and fallopian tubes differed significantly from those of the vagina. Furthermore, compared to the vagina and cervix, the ovaries, endometrium, myometrium, and fallopian tubes exhibited greater diversity in their respective microbial compositions (90). Chen et al. demonstrated that the microbial communities of the bilateral fallopian tubes were essentially identical, with characteristic Operational Taxonomic Units including Pseudomonas, Streptococcus, and Prevotella. Characteristic Operational Taxonomic Units in peritoneal fluid (PF) comprised Pseudomonas, Morganella, Sphingomonas, and Eubacterium (8). Pelzer et al. analyzed tubal microbial characteristics in patients undergoing total hysterectomy or salpingo-oophorectomy for benign or prophylactic conditions, revealing Staphylococcus as the most abundant genus in the fallopian tubes (91). Research indicates that postmenopausal women exhibit lower tubal bacterial diversity compared to premenopausal women. Furthermore, significant differences in tubal microbiota composition were observed between women with and without Mirena intrauterine devices, suggesting that hormonal changes may influence the tubal microbiome.

#### 5.2.5 Ovarian microbiota

Research on the ovarian microbiota remains particularly scarce, and microorganisms commonly found in follicular fluid can be isolated from the vagina, gastrointestinal tract, skin, and oral mucosa (92). Walther-António et al. conducted ovarian biopsies in patients undergoing total hysterectomy with bilateral salpingo-oophorectomy. In the benign disease group, the dominant genera of the ovarian microbiota were Oligotrophus and Lactobacillus, whereas Bacteroides dominated ovarian samples from the endometrial carcinoma group (93). Pelzer et al. found Lactobacillus and Propionibacterium to be the most prevalent genera in follicular fluid. Elevated levels of estrogen and P in follicular fluid may promote the growth of certain bacteria, such as Lactobacillus and Bifidobacterium. Studies have identified differences in bacterial species richness between right and left follicular fluids, potentially related to the independent blood supply of each ovary (94). The presence of Lactobacillus in ovarian follicular fluid correlates with embryo maturation and transfer, while high embryo transfer rates are associated with Lactobacillus presence in both ovarian follicular fluids (95). Wu et al. analyzed that follicular fluid in infertile patients was predominantly composed of Corynebacterium, Lactobacillus, and Gardnerella species (96). Wallace et al. observed reduced lactate levels in follicular fluid from cases where fertilized eggs failed to cleave or early cleavage-stage embryos failed to implant. Lactobacilli’s influence on female fertility may relate to their lactate-producing capacity (97). In normally ovulating individuals, insulin stimulates granulosa cells in the ovary to produce lactate. Conversely, granulosa cells from anovulatory patients exhibit insulin resistance (IR), leading to reduced lactate production. Lactate thus plays a significant role in follicular development (98).

### 5.3 Imbalance of the peritoneal microbiota and oral microbiota

Research on the relationship between PF microbiota and POI remains limited. Findings from Yuan et al. indicate that Pseudomonas and Acinetobacter are the most abundant microorganisms in PF (99). Ovarian reserve function is not only closely linked to the quantity and quality of a woman’s oocytes but also associated with conditions such as ovarian tumors and endometriosis. Untreated endometriosis may induce endocrine disorders and ovarian dysfunction. Analysis of α-diversity in the PF microbiota of endometriosis patients revealed no statistically significant differences compared to healthy controls. However, β-diversity analysis demonstrated statistically significant differences, with markedly increased abundance of Acinetobacter, Devosia, and Lactobacillus. Lee et al. observed markedly increased abundances of Acinetobacter, Pseudomonas, and Streptococcus within extracellular vesicles from the peritoneal fluid of patients with ovarian endometriosis, alongside significantly reduced abundances of Propionibacterium and Actinomyces (100). While alterations in the peritoneal fluid microbiota of endometriosis patients have been reported, the presence of specific pathogenic bacteria and the causal relationship between these microbial shifts and POI require further validation through research.

The oral cavity, serving as the common orifice for both the respiratory and digestive tracts, constitutes a highly complex ecosystem (101). The oral microbiome, a focal point of the Human Microbiome Project, is increasingly recognized for its association with systemic diseases (102, 103). Mounting evidence suggests a relationship between the oral microbiome and POI. Sex hormones may influence the composition of the oral microbiota. Postmenstrual women exhibit an increased abundance of actinobacteria in the oral cavity compared to premenstrual phases. Disruption of the salivary microbiota leads to dysbiosis of the GM, which in turn contributes to POI through gut microbial imbalance. Research indicates that the oral microbiome has the potential to predict adverse pregnancy outcomes (104). Wang et al., comparing oral, gut, and vaginal microbiomes between healthy pregnancies and gestational diabetes cases, found the most pronounced alterations in the oral microbiome. The abundance of oral Neisseria/Fimbriella species correlated positively with glucose levels in pregnant women, suggesting that the oral microbiota could serve as a biomarker for predicting pregnancy complications (105). The association between POI patients and the oral microbiome remains unexplored. Further prospective cohort studies are required to determine how the evolution of the oral microbiota under different conditions influences the progression of POI.

## 6 Mechanism of microbiota affecting POI

### 6.1 Microbial imbalance and HPO axis

The hypothalamus, pituitary gland, and ovaries influence one another through hormone secretion, forming a complete neuroendocrine regulatory system known as the HPO axis. Pulsatile secretion of GnRH from the hypothalamus signals to the pituitary gland, stimulating the release of gonadotropins (Gn), primarily FSH and LH. These hormones bind to corresponding receptors in ovarian tissue, regulating the growth and development of ovarian granulosa cells (106). Theca cells can secrete AMH and E2. AMH promotes the maturation of primordial follicular cells, while E2 is a crucial hormone for maintaining female reproductive health. Dysfunction of the HPO axis leads to abnormal hormone levels, which are detrimental to ovarian growth and development, ultimately resulting in POI. Microbiome imbalance increases local inflammatory factors. These inflammatory mediators and microbial metabolites enter the bloodstream, inducing chronic systemic inflammation that subsequently impacts the HPO axis and alters hormone levels (31). Research indicates that reduced levels of Bacteroides and Firmicutes may elevate serum GLP-1 concentrations. GLP-1 influences GnRH secretion by regulating gamma-aminobutyric acid (GABA) neurotransmission and kisspeptin neuron neurotransmission.

Microbiota metabolites SCFAs and BA directly regulate hypothalamic GnRH neurons. GnRH acts via the HPO axis on the ovaries, synthesizing and secreting P, E2, and testosterone, stimulating target organ development and influencing ovarian reserve function. SCFAs participate in the secretion of brain-gut peptides by enteroendocrine cells, which in turn regulate the hypothalamic regulatory nuclei and control LH secretion. SCFAs also inhibit excessive LH synthesis and release by attenuating the intensity of pituitary LH pulses. Microbiota dysbiosis induces insulin resistance and hyperinsulinemia, stimulating excessive ovarian androgen production (107). On one hand, elevated insulin levels heighten adrenal sensitivity to adrenocorticotropic hormone, increasing adrenal production of dehydroepiandrosterone and thereby elevating systemic androgen concentrations. While the ovaries, being insulin-sensitive target organs, experience impaired follicular development and ovulation due to high insulin and IR. This dual pathway stimulates both adrenal and ovarian androgen overproduction. Glucocorticoids and androgens interact reciprocally, with androgens influencing early follicular development. Research indicates that moderate testosterone levels effectively enhance ovarian reserve function (108).

### 6.2 Microbial imbalance exacerbates endocrine metabolic imbalance

Ovarian endocrine levels indirectly reflect ovarian function (109). As an ‘endocrine organ’, disruption of the microbiome’s equilibrium disturbs the body’s internal homeostasis, leading to disordered secretion of steroid hormones, insulin, and other substances, thereby increasing the risk of reproductive system disorders (110). The GM maintains the integrity of the intestinal barrier, preventing bacteria and metabolic products from entering the bloodstream and thus avoiding interference with sex hormone function (111). GM and its metabolites also improve ovarian function and oocyte quality by regulating glucose and lipid metabolism (112). SCFAs influence hormone secretion by activating intestinal GPCRs (113). Butyrate activates steroid hormone synthesis (including P and E2 in ovarian granulosa cells via the cAMP signaling pathway and increases histone H3K9 acetylation (114). Butyrate enhances lipogenesis through multiple mechanisms, including upregulating glucose uptake and lipogenesis, and inducing glucose transporter four and PPAR-γ, thereby improving apoptosis and glucose metabolism in human ovarian granulosa cells (115). The microbiota communicates with the vagus nerve by producing neurotransmitters, such as serotonin, thereby influencing hypothalamic neuroendocrine cells and regulating sex hormone secretion (116). Concurrently, the GM participates in the metabolism of essential nutrients, including vitamin D and B vitamins, which are crucial for sex hormone synthesis and reproductive health.

The microbiota contributes to regulating estrogen levels (117, 118). When E2 secretion is insufficient and FSH secretion is excessive, a preliminary assessment of ovarian reserve function is vital for diagnosing POI (119). β-glucuronidase secreted by bacteria such as Bifidobacterium and Escherichia coli constitutes a key factor in modulating circulating estrogen concentrations (120). Research indicates that Klebsiella aerogenes in the feces of premenopausal women can produce 3β-hydroxysteroid dehydrogenase, which degrades E2, leading to reduced estrogen levels (121). Changes in estrogen levels further influence GM homeostasis, with the relative abundance of Proteobacteria positively correlated with E2, while that of the Prevotellaceae family negatively correlated with E2 (64). Gajer et al. observed that the composition and abundance of the vaginal microbiota are primarily influenced by estrogen and P, with this influence commencing during puberty and maintaining a dynamic equilibrium throughout the reproductive years (122). Teixeira et al., investigating the relationship between Lactobacillus and Gardnerella in germ-free mice, found that estrogen is an essential factor for Lactobacillus colonization (123). Hyman et al. examined estrogen levels and vaginal microbiota composition at different stages in women undergoing IVF-ET treatment. They observed a significant increase in estrogen levels from the initial follicular to the mature follicular phase in all patients, with over half exhibiting concomitant changes in vaginal microbiota. From human chorionic Gn injection to embryo transfer, patients experienced a sharp decline in estrogen levels, consistently accompanied by alterations in vaginal microbiota, a correlation between vaginal microbiota changes and estrogen levels, with Lactobacillus dominance on the transfer day being a critical factor for IVF-ET success (124). Consequently, targeted microbiota supplementation may correct endocrine-metabolic imbalances, offering a highly promising novel therapeutic strategy for POI. Estrogen plays important regulatory role in the maintenance of the immune system functions and to counteract inflammations and oxidative stress (125).

### 6.3 Microbial imbalance exacerbates inflammation

Microbiome imbalance may interfere with ovulation by mediating inflammatory responses (126). In 2009, Round et al. indicated that GM dysregulation compromises intestinal barrier function, increasing intestinal permeability and allowing bacteria and their metabolites to enter the bloodstream, thereby triggering systemic inflammatory responses. This inflammatory environment disrupts the production and regulation of sex hormones, impairing reproductive function (127). Alterations in microbial composition may also influence ovarian inflammation, modifying ovarian gene expression and leading to diminished oocyte quality (128). Diet-induced alterations in GM composition may trigger ovarian inflammatory responses, subsequently modifying ovarian gene expression, ultimately resulting in diminished oocyte quality and infertility in obese individuals. Increased levels of vaginal chemokines, such as IL-8 and IL-18, correlate with specific vaginal microbiota species, with IL-8 levels exhibiting a negative correlation with lactobacilli abundance (94). Overgrowth of anaerobic bacteria produces toxic substances that trigger the release of pro-inflammatory cytokines IL-1β and IL-8, concurrently reducing Lactobacillus levels and stimulating cytokine-and-chemokine signaling cascades. Conversely, the microbiota and its metabolites may also improve ovarian function and reduce inflammation by mediating inflammatory cytokine expression (129). SCFAs protect mice from inflammatory damage by interacting with GPCR4, regulating cellular metabolism, and inhibiting histone deacetylases, thereby promoting IL-22 production by CD4+ T cells (130). High-fat diet-induced GM dysbiosis in mice led to increased Gram-negative bacteria, heightened endotoxin production, and promoted ovarian macrophage infiltration, thereby inducing inflammatory responses. Transplanting GM from high-fat diet mice into normal mice also exhibited ovarian inflammation. Conversely, transplanting normal mouse fecal microbiota into mice alleviated ovarian inflammatory responses (131). Research indicates that dihydroxyflavone intervention effectively prevents follicular atresia, safeguards healthy oocyte development, elevates serum E2 levels, and reduces urinary FSH levels. Its mechanism of action may involve modulating the HPO axis by attenuating GM-mediated inflammatory responses. Jinfeng Pill is effective to ameliorate the symptoms of POI induced by CTX in the model of rats and its action is likely associated with suppressing IL-17A/IL-6 axis and the activity of MEK1/2-ERK1/2 signaling (132). The inflammatory microenvironment in POI applies to many levels (133). Consequently, regulating the microbiota to mitigate inflammatory responses may offer novel therapeutic strategies for enhancing reproductive health.

### 6.4 Microbial imbalance exacerbates immune imbalance

Immune-inflammatory responses are closely associated with the onset of POI (134). Within the ‘microbiome-ovarian axis’, immune regulatory pathways form the core hub linking microbial signals to ovarian function. These pathways constitute a multi-system immunological dialogue network, meticulously programmed by microbial metabolites. Their central mechanism involves the microbiome exerting both systemic “regulation” and localized ‘fine-tuning’ of the host immune system through its ‘metabolic reservoir’, ultimately determining the immune homeostasis of the ovarian microenvironment and follicular fate (135). Studies indicate that patients with POI exhibit autoimmune ovarian inflammation, with histological examination of ovarian tissue revealing extensive infiltration by inflammatory cells, including B cells, T cells, and mononuclear macrophages, leading to ovarian atrophy and functional failure (136, 137). Altered levels of IL-2 and IFN-γ in peripheral blood persistently activate cytokines such as TNF-α and IL-1, promoting B-cell proliferation, differentiation, and antibody secretion. This induces an autoimmune response, accelerating the loss or apoptosis of ovarian antigen-targeted cells and hastening follicular atresia (138).

Microbiome dysbiosis induces excessive immune-inflammatory responses in ovarian tissue, impairing ovarian function (139). GM maintains intestinal barrier function and regulates intestinal permeability, preventing pathogens and harmful substances from entering the bloodstream and thereby reducing inflammation and autoimmune reactions (140). Research indicates that GM synthesizes intestinal mucosal secretory immunoglobulins and protects ovarian function by regulating immune cytokines, including regulatory T cells (Treg), Th17 cells, and IFN-γ (141). SCFAs enhance GM abundance, increase Treg expression and differentiation, and reduce Th17 differentiation. The equilibrium between Th17 and Treg cells is crucial for maintaining ovarian function (142). Yin et al. observed altered Treg numbers and improved immunoregulatory function in POF mice following treatment (143). GM therapy influences serum IFN-γ levels, in which IFN-γ promotes MHC class II antigen expression, stimulates autoimmune responses, and induces follicular atresia, leading to POF. Jiang et al. further investigated HRT’s impact on POI, observing that in POI patients undergoing HRT, the previously increased fecal abundance of Clostridium difficile and elevated serum transforming growth factor-β1 levels were reversed (144). Thus, preventive interventions targeting the microenvironment and enhancing local immune function may offer novel insights for delaying POI progression and its management.

### 6.5 Microbial imbalance exacerbates oxidative stress

Ovarian dysfunction is closely associated with mitochondrial dysfunction, and oxidative stress-induced mitochondrial dysfunction constitutes a significant factor in POI (145, 146). Oxidative stress, arising from excessive ROS production and defects in antioxidant defense mechanisms, is considered the primary cause of granulosa cell apoptosis (147). Within ovarian tissue, normal levels of ROS play a crucial regulatory role in follicular growth, intrasheath angiogenesis, and sex hormone synthesis. However, elevated ROS levels cause cellular damage, manifesting as impaired follicular and oocyte development, hormonal dysregulation, and reduced fertility (148). Oxidative stress injury downregulates antioxidant gene expression, leading to ovarian lipid peroxidation and DNA oxidative damage, which impairs ovarian function and oocyte quality (149). Reducing oxidative stress in ovarian granulosa cells mitigates ovarian damage, reduces follicular atresia, and delays the progression of POI (150).

Research indicates that flavonoids and phenolic compounds secreted by Lactobacillus in bovine follicular fluid possess antioxidant properties, protecting oocytes from oxidative stress, reducing arachidonic acid secretion in follicular fluid, and improving oocyte quality (97). Compared to women with regular menstrual cycles, anovulatory women with irregular cycles exhibit significant differences in GM composition, characterized by a lower abundance of butyrate-producing bacteria. Butyrate possesses antioxidant effects; its deficiency may foster a microinflammatory environment, contributing to ovulatory dysfunction (151). Research indicates that supplementation with branched-chain amino acids (BCAAs) modulates ovarian function and fertility via the ceramide-ROS axis. Dietary BCAAs protect mouse ovaries from ROS-induced POI (152).

## 7 Potential treatments for microbiome-based therapies in POI

### 7.1 Diet regulation

Through lifestyle modifications, supplementation with probiotics, and targeted microbiota therapies, it is possible to effectively modulate microbial composition and enhance female reproductive health (153). Given that obesity, excessive body fat, and insulin resistance adversely affect follicular development, thereby impairing female fertility (154). Consequently, restricting caloric intake can reduce body weight, decrease visceral abdominal fat accumulation, and enhance insulin sensitivity, thereby improving ovarian function and delaying ovarian ageing (64). Lifestyle modifications, including dietary changes, regular exercise, and stress management, can significantly influence microbial diversity (155). Consuming foods rich in fiber, fruits, vegetables, and fermented products promotes gut diversity by fostering the growth of beneficial bacteria such as Lactobacillus and Bifidobacterium (156). These beneficial bacteria produce SCFAs, which exert anti-inflammatory effects and maintain intestinal barrier integrity. Omega-3 polyunsaturated fatty acids, as essential fatty acids for humans, play a role in regulating intestinal immune function and maintaining gut homeostasis (157, 158). Women with higher intakes of fruit, vegetables, dairy products, or omega-3 polyunsaturated fatty acids exhibit a relatively lower risk of endometriosis (159). Furthermore, regular physical exercise has been demonstrated to promote GM diversity and reduce inflammation, which are crucial for maintaining reproductive health.

### 7.2 Microecologics

Probiotics can effectively inhibit the growth of pathogenic bacteria, thereby maintaining the balance of the intestinal microflora (160). Supplementation with specific probiotics, such as lactobacilli and bifidobacteria, has been demonstrated to enhance gut health and improve immune function, thereby benefiting reproductive health (161). Research by Calcaterra et al. revealed that probiotic supplementation can improve hormone levels, reduce inflammatory markers, and correct lipid metabolism disorders in patients with polycystic ovary syndrome (PCOS). Early adolescent supplementation may prevent PCOS onset and protect fertility (162). Chadchan et al. indicated that probiotics may improve gut health, reduce inflammation, and potentially benefit ovarian function (163). Prebiotics are organic substances that are not digested or absorbed by the host but selectively promote the metabolism and proliferation of beneficial bacteria within the body, thereby improving host health (164). Synbiotics, as composite formulations combining probiotics and prebiotics, have also demonstrated therapeutic effects similar to those of probiotics alone (165). Chenoll E et al. isolated the strain Lactobacillus rhamnosus BPL005 from vaginal samples and co-cultured it with cell models of Atopobium vaginae, Propionibacterium acnes, and Streptococcus agalactiae, revealing a marked reduction in Propionibacterium acnes and Streptococcus agalactiae levels (166). These studies indicate the potential application of microbial preparations in reproductive health, offering a novel therapeutic approach for POI.

### 7.3 Microbiome transplantation

**Figure 3:**
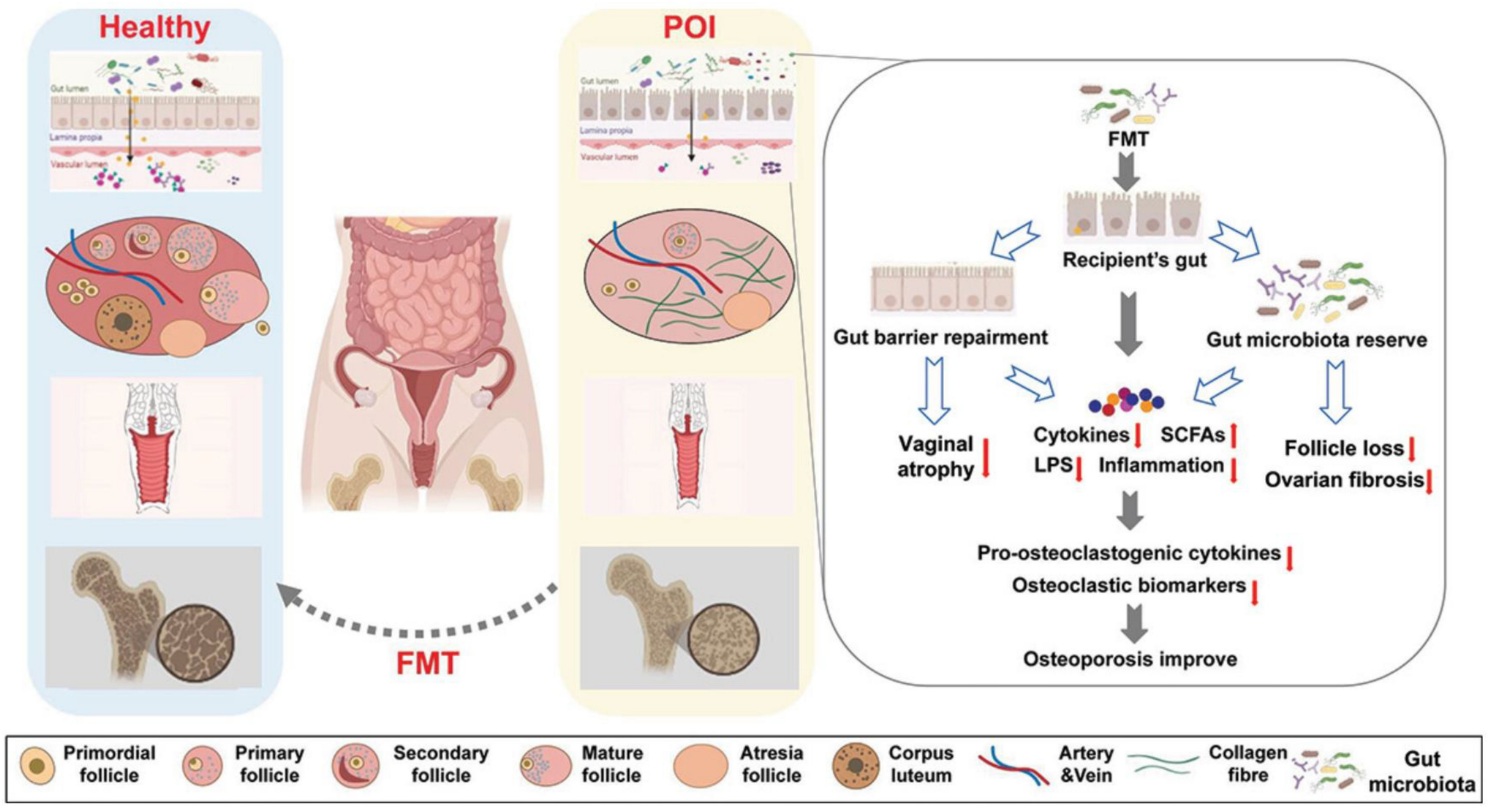
FMT alter the gut microbiota and slow the progression of ovarian aging related diseases (64).

Microbiome transplantation primarily refers to the transfer of microbial material from a healthy donor to a recipient and represents a novel therapeutic approach for restoring microbial balance (167). Lev-Sagie et al. observed that vaginal microbiome transplantation achieved long-term complete remission of bacterial vaginosis-associated symptoms, with the dominant vaginal microbiota restored to Lactobacillus species, offering new prospects for future research into FRT microbial dysbiosis (168). Li et al. transplanted GM from 5-week-old mice into 42-week-old mice, observing that the young microbiota remodeled the immune microenvironment of ageing ovaries. This suggests Fecal microbiota transplantation (FMT)delays ovarian ageing via dual pathways of ‘anti-inflammation and immune modulation’ (48). Research by Singh et al. revealed that FMT not only mediates inflammatory cytokine release but also regulates GM diversity, blood glucose levels, and insulin sensitivity. Following FMT treatment in PCOS rats, increased abundances of Lactobacillus and Clostridium, alongside reduced Prevotella, were observed, accompanied by improved oestrous cycles, decreased androgen biosynthesis, and normalized ovarian morphology (169). In mice with endometriosis, FMT treatment reduced the growth of endometriotic lesions (163). Zhang et al. demonstrated for the first time that GM could improve semen quality and spermatogenesis, treating busulfan-induced male infertility, by transplanting fecal microbiota from mice consuming alginate oligosaccharides (170). FMT can also utilize 3-hydroxyphenylacetic acid produced by GM to mitigate age-related spermatogenic dysfunction and restore spermatogenesis (171). Although no reports exist on microbiota transplantation in POI patients, this technique may be considered a future therapeutic approach to restore ovarian function **Figure 3** (64).

## 8 Conclusion

The high incidence and low cure rate of POI pose evident hazards to women. The microbiome is intrinsically linked to POI development **Table 1**, making the maintenance of a healthy microecological environment crucial for its prevention. Microbiota and their metabolites influence the reproductive system through pathways regulating the HPO axis, endocrine metabolism, inflammatory responses, immune responses, and oxidative stress. These mechanisms interact synergistically, collectively impacting female reproductive health via the ‘microbiome-ovarian axis’. With advancing microbiome research, integrating pharmacotherapy with probiotics—combining ‘microbiome therapy’ with conventional HRT—achieves synergistic effects while mitigating HRT side effects. This approach offers novel diagnostic and therapeutic avenues for POI, expanding possibilities for affected women. The microbiome holds promise as a therapeutic target for reproductive disorders, though several challenges remain: ① Identifying which microbial communities indicate ‘microbiome dysregulation’ in the FRT. ② Microbiome metabolites influence the synthesis and metabolism of substances such as glucose, lipids, and hormones through various signaling pathways, thereby affecting ovarian function. However, the mechanisms by which these metabolites directly impact germ cells or granulosa cells require further investigation. ③ Establishing a causal relationship between microbiota and POI is challenging due to the interactive nature of potential pathogenic factors. ④ How the high degree of individualization within the microbiota informs the universality of POI treatment. Moving forward, interdisciplinary collaboration between microbiologists, reproductive endocrinologists, immunologists, and bioinformaticians will be the sole pathway to unravel this complex network and ultimately deliver novel solutions for POI patients.

**Table 1:** Changes in the microbiota associated with ovarian dysfunction.

Disease	Sample source	Main findings	References
ovariectomy	Mice	Gnotobiotic mice that received the gut microbiome from ovariectomized mice fed the low-fat diet had greater weight gain and hepatic gene expression related to metabolic dysfunction and inflammation than those that received intact sham control-associated microbiome.	(25)
ovariectomy	Mice	Microbiota-based intervention to delay or reserve ovarian aging is an appealing approach and may offer new therapeutic strategies for intestinal microbiota regulation to improve female fertility.	(64)
POI	human	*E. hallii* and *E. ventriosum* have protective effects against POI, whereas *Intestinibacter* and *Terrisporobacter* have detrimental effects on POI	(65)
POF	Mice	the proportions of Helicobacter, Odoribacter, and Alistipes were lower in the dominant flora of the POF group, while Clostridium XIVa, Barnesiella, Bacteroides, and Mucispirillum were higher	(66)
POI	human	phylum Bacteroidetes, genera Butyricimonas, Dorea, Lachnobacterium and Sutterella enriched significantly in women with POI	(67)
POI	human	Bacteria in the genera Lactobacillus, Brevundimonas, and Odoribacter were more abundant in the microbiomes of healthy women, while the quantity of bacteria in the genus Streptococcus was significantly increased in the microbiomes of women with POI.	(68)
POF	human	The diversity and richness of the vaginal flora of patients with POF was significantly different from those of healthy controls. The relative abundance of L. gallinarum in the vagina was correlated with the FSH, E2 and AMH levels.	(71)
POI	human	Actinobacteria, Atopobium, and Gardnerella were significantly increased in POI patients	(76)
POF	Mice	During CTX induced POF, the abundance of Akkermansia decreased while the abundance of Lactobacillus increased	(130)
POI	human	higher *Butyricimonas*, *Dorea*, *Lachnobacterium*, and *Sutterella* and lower *Bulleidia* and *Faecalibacterium* abundances at the genus level were observed in women with POI	(144)
POF	human	POF patients showed significantly lower levels of Lactobacillus, Bifidobacterium, and gmGUS than controls.	(172)
